# Seasonal variations of the sea surface microlayer at the Boknis Eck Times Series Station (Baltic Sea)

**DOI:** 10.1093/plankt/fbx055

**Published:** 2017-10-04

**Authors:** Alexander Dreshchinskii, Anja Engel

**Affiliations:** 1 GEOMAR Helmholtz Centre for Ocean Research Kiel, Duesternbrooker Weg 20, 24105 Kiel, Germany

**Keywords:** sea surface microlayer, carbohydrates, amino acids, TEP, CSP, Time Series Station, plankton blooms

## Abstract

The sea surface microlayer (SML) is the uppermost layer of the water column that links the ocean and atmosphere. It accumulates a variety of biogenic surface-active and buoyant substances, including gelatinous material, such as transparent exopolymer particles (TEP) and Coomassie stainable particles (CSP), potentially affecting air–sea exchange processes. Here, we studied the influence of the annual cycle of phytoplankton production on organic matter (OM) accumulation in the SML relative to the subsurface water (SSW). Sampling was performed monthly from April 2012 to November 2013 at the Boknis Eck Time Series Station (Baltic Sea). For SML sampling, we used the Garrett screen, while SSW samples were collected by Niskin bottles at 1 m depth. Samples were analyzed for carbohydrates, amino acids, TEP, CSP, chlorophyll *a* (SSW only) and bacterial abundance. Our data showed that the SML reflected the SSW during most parts of the year, with changes mainly responding to bloom formation and decay. OM composition during phytoplankton blooms clearly differed from periods of higher bacterial abundance. Of all components investigated, only the enrichment of total carbohydrates in the SML was inversely related to the wind speed indicating that wind-driven mixing also affected the accumulation of OM in the SML during our study.

## INTRODUCTION

The sea surface microlayer (SML) is the thin boundary layer between the ocean and the atmosphere and chemically and is physically different from the subsurface water (SSW). It is formed by a variety of surface-active and buoyant organic compounds that tend to accumulate at the air–sea interface (Cunliffe *et al.*, 2011). Carbohydrates and amino acids have been shown to be major biochemical components of the SML (Sieburth *et al.*, 1976; Henrichs and Williams, 1985; Kuznetsova *et al.*, 2004) and can accumulate as gelatinous material. This includes the polysaccharidic transparent exopolymer particles (TEP) and proteinaceous Coomassie stainable particles (CSP) providing a complex gel-like nature of SML (Wurl and Holmes, 2008; Cunliffe and Murrell, 2009). It is considered that gels are produced directly by phytoplankton (Long and Azam, 1996; Passow, 2002b), or form by assembly (Chin *et al.*, 1998) and subsequent coagulation from low molecular weight precursors (Engel *et al.*, 2004). Coagulation presumably favors the enrichment of gel particles in the SML during surface wind shear (Wurl *et al.*, 2011a). In addition, positive buoyancy of gels (Azetsu-Scott and Passow, 2004; Mari *et al.*, 2017) and the adsorption of their precursors onto rising air bubbles, (Zhou *et al.*, 1998) can also lead to the accumulation of gels in the SML.

While the accumulation of organic material in the SML often reflects phytoplankton bloom dynamics in SSWs (Žutić *et al.*, 1981; Gao *et al.*, 2012; Engel *et al.*, 2014), other factors, including wind speed, irradiance and bacterio- and phytoneuston activity, also influence the SML chemical composition. Increasing wind speed is thought to disperse particulate organic components from the SML into the bulk water, thereby causing their transient depletion in the microlayer (Liu and Dickhut, 1998; Obernosterer *et al.*, 2008; Wurl *et al.*, 2009, 2011b; Engel and Galgani, 2016). However, surface-active compounds have been recently shown to accumulate in the Atlantic Ocean microlayer up to wind speeds of 13 m s^−1^ (Sabbaghzadeh *et al.*, 2017). Bacterial degradation apparently results in the removal of organic material from the SML as well, but bacteria also release organic polymers further contributing to the SML organic matter (OM) pool (Decho, 1990; Stoderegger and Herndl, 1999; Radić *et al.*, 2006; Sugimoto *et al.*, 2007). Solar radiation exerts additional influence on the SML, inducing various photochemical reactions that can both modify and degrade its components (Plane *et al.*, 1997).

The SML is considered as crucial for the exchange of climate relevant gases between the ocean and atmosphere, as it may alter the rate of gas transfer by wave damping and by reducing turbulent exchange (Frew *et al.*, 1990; Liss and Duce, 1997; Salter, 2010). Variations in SML composition can therefore potentially cause changes in hydrodynamic processes at the air–sea interface and therewith gas transfer rate. So, their understanding would be required to relate physical and chemical processes at the air–sea interface to ecosystem dynamics, which are responding to a changing environment.

Previous studies predominantly covered short-term variations in biochemical components of the SML (Henrichs and Williams, 1985; Falkowska, 1999; Kuznetsova *et al.*, 2004; Reinthaler *et al.*, 2008; Galgani and Engel, 2013; Galgani *et al.*, 2014), while long-term variations remain largely unknown.

The objective of this study was to investigate the accumulation and composition of OM within the SML over a full annual cycle. We hypothesized that the organic composition of the SML is dynamically influenced by seasonal changes in the SSW ecosystem and reflect variations in the dominance of phyto- and bacterioplankton. Our study was conducted at the Boknis Eck Time Series Station, a temperate monitoring site operated since 1957. Clear seasonal patterns of pelagic system characterized by pronounced phytoplankton bloom stages in spring and autumn are regularly observed at Boknis Eck. Here, we studied the accumulation of high molecular weight polysaccharides, combined amino acids and gelatinous material, including TEP and CSP, referring each of these parameters to the corresponding values in the upper water column.

## METHOD

### Sampling area

**Fig. 1.**
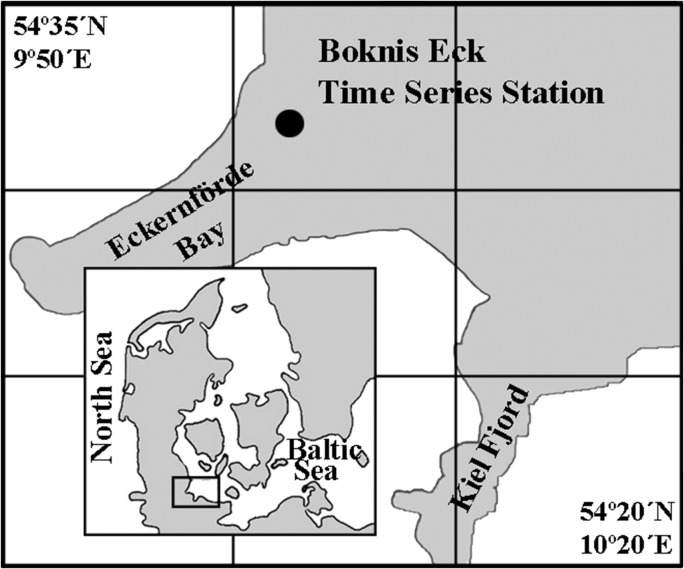
Location of the Boknis Eck Time Series Station in the southwestern Baltic Sea.

Sampling was performed at the Boknis Eck Time Series Station (www.bokniseck.de) located near the entrance of the Eckernförde Bay (54°31′N, 10°02′E) in the southwestern Baltic Sea, ~1 nautical mile off the coast (Fig. 1). Water conditions at Boknis Eck are typical for the southwestern Baltic Sea region and are characterized by water masses inflow from the brackish Baltic and more saline North Sea with little riverine inputs (Lenz, 1977a). From March to September, there is a pronounced temperature and salinity stratification of the water column, with averaged thickness of the surface mixed layer of 10–15 m (Hoppe *et al.*, 2013). As typical for a temperate coastal system, the plankton community shows a clear seasonal cycle with pronounced phytoplankton blooms occurring in spring (February–March) and in autumn (September–November).

### Seawater sampling

Samples of the SML and SSW were collected monthly from April 2012 to November 2013 around the noontime from the weatherboard of the research vessel *Littorina*. SML sampling was conducted after Garrett (1965) using a stainless-steel screen (14-mesh sieve of 30 cm in diameter, Linker Industrietechnik), similar to the 16-mesh sieve suggested by the Intergovernmental Oceanographic Commission (IOC, 1985) as a standard tool for SML sampling. The main reason for choosing the Garrett screen was the weather condition in the study area, with strong winds (up to 15 m s^−1^) encountered over the year. The screen technique allows for collecting a relatively large amount of samples in a short period of time suitable even at the high wind speeds (Cunliffe and Wurl, 2014). Briefly, the screen was submerged and withdrawn horizontally through the sea surface before being drained into a sample bottle (250 mL or 500 mL Schott Duran Laboratory glass bottles). Typically, 3–4 dips were needed to fill a 250 mL bottle corresponding to a thickness of the sampled microlayer of ~300–400 μm (the thickness is the ratio of the water volume collected to the surface area of the screen). SSW samples were taken at the same location from 1 m depth using 5 L Niskin bottles mounted to a Seabird CTD. In total, ~2.0 L of SML and 1.5 L of SSW were collected during each sampling. Prior to sampling, the screen was washed in the dishwasher and rinsed copiously with surface seawater immediately before sampling.

The samples were placed cold (~4°C) and in the dark for ~3 h while carried to the home laboratory. Subsamples for dissolved carbohydrates and amino acids were filtered (see below) in the ship's laboratory immediately after sampling. All glass equipment involved in sample storage was previously cleaned by washing with 10% HCl and rinsing with ultrapure Milli-Q water (Elga).

### Chemical and biological analyses

TEP were quantified both microscopically (Engel, 2009) and colorimetrically (Passow and Alldredge, 1995). Subsamples of 20–60 mL were filtered onto 25 mm 0.4 μm polycarbonate filters (Nuclepore) under low pressure (<200 mbar). Staining of the retaining particles was conducted by adding 1 mL of filtered (<0.2 μm) Alcian Blue solution to the filter for ~3 s. After rinsing with ultrapure Milli-Q water, the filters were transferred either to polypropylene tubes or to semi-permanent Cytoclear© slides and stored frozen at −20°C. All samples were analyzed in duplicate. For colorimetric analysis, 6 mL of 80% sulfuric acid was added into each polypropylene tube to allow the dye migration from the filter. After 2.5 h, the released amount of Alcian Blue was measured spectrophotometrically at 787 nm in a 1 cm cuvette. Alcian Blue was calibrated with Xanthan gum (Fluka Biochemica); TEP concentrations were expressed as Xanthan gum equivalent per liter (μg Xeq. L^−1^). The calibration factor changed three times during the study period, being 149 ± 22 from April to December 2012, 165 ± 24 from February to July 2013 and 158 ± 24 from August to November 2013. Stained TEP were examined under a compound light microscope equipped with a digital AxioCam MRc camera (Zeiss) at 200× magnification. About 30 images per filter were taken in a cross-section. Enumeration and sizing of gel particles (>0.2 μm^2^) was carried out with the aid of the image analysis software WCIF ImageJ.

CSP were determined microscopically according to Engel (2009). Filters were prepared in the same manner as described above for TEP except that the staining stage was conducted with Coomassie Brilliant Blue for 30 s.

Both TEP and CSP filter blanks were prepared with Milli-Q water processed as seawater samples. The standard deviations between the replicate filters were generally <30% for the microscopic parameters (the total area and the particle abundance), with higher values (50%) for few replicates. The replicates of the colorimetric method agreed within 25%.

For bacterial cell counts, 4.5 mL subsamples were fixed with 200 μL glutaraldehyde 25% solution and then stored at −20°C until analysis. Bacterial cells were detected by flow cytometry following Gasol and del Giorgio (2000). A 200 μL thawed subsample was stained with 10 μL SYBR Green (Invitrogen) stock solution (20% SYBR Green in dimethyl sulfoxide (Sigma-Aldrich)) for 10 min at room temperature in the dark. Fluoresbrite latex beads (0.94 μm, Polysciences) were used as internal standard. After adding 10 μL beads to each stained sample the latest were run at a low flow rate through a FACS Calibur flow cytometer (BD, Becton Dickinson). TruCount beads (BD) served for calibration. Finally, bacterial enumerations were done with the aid of the software “Cell Quest Pro” (BD Biosciences). The standard deviations for this analysis generally lay below 20%.

Carbohydrates (CHO) were determined by ion chromatography according to Engel and Händel (2011). For high molecular weight (>1 kDa) dissolved combined carbohydrates (DCCHO), 15 mL samples were filtered through 0.45 μm syringe-filters (GHP membrane, Acrodisk, Pall Corporation) and stored in precombusted (8 h at 500°C) glass vials at −20°C. For total combined carbohydrates (TCCHO), the same amount of each sample was frozen unfiltered. Prior to analysis, 7 mL subsamples were desalted by membrane dialysis using dialysis tubes with 1 kDa molecular weight cut-off (Spectra Por). The desalination was conducted for 4.5 h at 1°C. Thereafter, 2 mL duplicate subsamples were sealed with 1.6 mL 1 M HCl in precombusted (8 h at 500°C) glass ampoules and hydrolyzed to obtain monomeric CHO. Hydrolysis was done for 20 h at 100°C where upon the ampoules were cooled to a room temperature and stored frozen at −20°C until further processing (within 5 months). Finally, the subsamples were neutralized by acid evaporation under N_2_ atmosphere at 50°C and resuspended with ultrapure Milli-Q water. Determination of CHO monomers was carried out by high-performance anion exchange chromatography (HPAEC) coupled with pulsed amperometric detection (PAD) on a Dionex ICS 3000 system. Their separation was achieved by a Dionex CarboPac PA10 analytical column (2 × 250 mm) coupled to a Dionex CarboPac PA10 guard column (2 × 50 mm). This method allows measurement of fucose (Fuc), rhamnose (Rha), galactosamine (GalN), arabinose (Ara), glucosamine (GlcN), galactose (Gal), glucose (Glc), combined mannose and xylose (Man/Xyl), gluconic acid (GlcA), galacturonic acid (Gal-URA) and glucuronic acid (Glc-URA). Due to coelution, mannose and xylose were measured as the sum of each other. The standard deviations of replicates were typically <20% for the majority of individual CHO, except galactosamine which diverged by 40% in total and by 30% in dissolved fractions.

The ratio of the amino sugars glucosamine and galactosamine (GlcN:GalN) was calculated as it may indicate the potential source of these compounds (Müller *et al.*, 1986; Ogawa *et al.*, 2001; Benner and Kaiser, 2003). Thus, a bacterial source of organic material is characterized by relatively low (<2) GlcN:GalN values, whereas high values (>8) generally indicates an origin from phyto- and zooplankton.

Amino acids were determined by high-performance liquid chromatography (HPLC) following the approach described by Lindroth and Mopper (1979). Of note, 6 mL seawater samples for total and dissolved hydrolysable amino acids (THAA and DHAA, respectively) were prepared and stored identically to those destined for carbohydrate. For hydrolysis, 0.5 mL thawed subsamples were transferred into precombusted (8 h at 500°C) glass ampoules and mixed with 0.5 mL HCL (6 M). After flushing with N_2_, the ampoules were sealed and placed at 110°C for 24 h. The hydrolyzates were then dried under N_2_ atmosphere with the following resuspension in ultrapure Milli-Q water (repeated two times). Precolumn amino acids derivatizaton with o-phthaldialdehyde was used to obtain fluorescent derivatives of the individual amino acids, which were then separated on a Kinetex (Phenomenex) C_18_ 150 mm-length column (2.7 μm particles). The eluent composed of the binary solvent system with (A) 0.01 M monosodium phosphate adjusted with NaOH to pH 7 and (B) acetonitrile. Asparagine and glutamine were deaminated by the hydrolysis and were quantified as aspartic acid (AsX) and glutamic acid (GlX). Glycine (Gly), serine (Ser), alanine (Ala), tyrosine (Tyr), valine (Val), isoleucine (Iso), phenylalanine (Phe), AsX, GlX and leucine (Leu) replicates agreed within 10%, except threonine (Thr), arginine (Arg) and γ-aminobutyric acid (GABA), with the standard deviations <20%.

Samples for chlorophyll *a* (Chl *a*) were collected as part of the monitoring program and measured using the fluorometric method of Welschmeyer (1994). Briefly, samples for Chl *a* were vacuum-filtered (<200 mbar) onto Whatman GF/F filters (25 mm, 0.7 μm) whereupon Chl *a* was extracted in 90% acetone in the dark and analyzed on a Trilogy® fluorometer (Turner Designs). Prior to analysis, the fluorometer was calibrated with a Chl *a* standard (*Anacystis nidulans*, Walter CMP, Kiel, Germany).

All data are available at the PANGAEA database (www.pangaea.de).

### Physical parameters measurement

The wind speed data were obtained by measurements from the research vessel anemometer during each sampling.

The data on temperature and salinity were obtained from the CTD equipped with a Temperature and Salinity sensor Typ CT_/494 (Sea & Sun Technology).

### Calculations and statistical analysis

The extent of the SML enrichment in organic material relative to the SSW, i.e. the enrichment factor (EF), was estimated by the ratio of the compound parameter in the microlayer to that in SSW.

Calculations and statistical tests were performed with Microsoft Office Excel 2010 equipped with statistics Add-In WinSTAT. Mann–Whitney *U*-tests were applied to assess statistical differences between the SML and SSW components as well as to statistical comparison of EFs with unity. We considered that null hypotheses testing and correlations were significant at *P* < 0.05. Results are presented as mean value ±1 standard deviation (SD).

Principal component analyses (PCAs) were done with NCSS 2004 software package (NCSS, LLC, Kaysville, Utah, USA). PCA is a multivariate regression analysis that allows the description of multivariable data by means of a few number of latent variables called principal components. This method is frequently used to determine compositional trends in seawater and sediment OM, in particular, to explore the extent of sample degradation (Dauwe and Middelburg, 1998; Amon *et al.*, 2001; Goutx *et al.*, 2007). In this study, eight separate PCAs were performed on carbohydrates and amino acid data sets of microlayer and SSW. Prior to analyses, the original data sets were centered and scaled by subtracting the average and dividing by the standard deviation.

## RESULTS

### Temporal development of environmental conditions, chlorophyll *a* and bacterial abundance

**Fig. 2.**
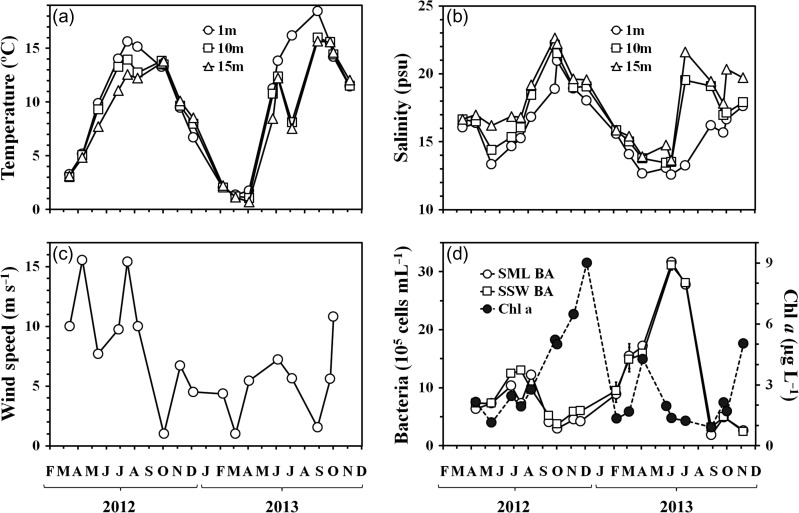
Variations of environmental parameters and organic components in the SML and SSW during the study period (April 2012–November 2013) (**a**) temperature and (**b**) salinity at 1 m (SSW), 10 m and 15 m depth; (**c**) the wind speed; (**d**) chlorophyll *a* concentrations (only in SSW) and bacterial abundance.

The temperature and salinity profiles (Fig. 2a and b) indicated that the first 15 m of the water column were homogenously mixed throughout winter (October 2012–March 2013). Over the rest of the study period, water column stratification occurred, and was strongest between May and September. In the SSW, i.e. at 1 m, the temperature varied from 1.4 to 18.5°C and the salinity varied from 12.6 to 21.0 psu, a typical situation for Boknis Eck (Hoppe *et al.*, 2013). Both temperature and salinity showed a pronounced seasonality. Statistically, changes in SSW temperature significantly lagged from changes in salinity by 2 months (*r*^2^ = 0.62, *P* < 0.005). Wind speed varied from 1.0 to 15.6 m s^−1^, with an overall average of 7.0 ± 4.4 m s^−1^ (Fig. 2c). Strongest winds (11.7 ± 3.6 m s^−1^) occurred during sampling from April to August 2012, while calmer conditions (4.3 ± 2.7 m s^−1^) were met on most other sampling days, with periods of particularly low wind speed (<1.6 m s^−1^) in October 2012, March and August 2013.

Chlorophyll *a* (Chl *a*) concentration in the SSW ranged from 1.5 to 9.0 μg L^−1^ (Fig. 2d), with highest concentrations in September–December 2012 (up to 9.0 μg L^−1^) and in April 2013 (up to 4.3 μg L^−1^), indicating the appearance of pronounced autumn and spring phytoplankton blooms. Bacterial abundance ranged from 1.9 to 31.7 × 10^5^ cells mL^−1^ both in the SML and in the SSW. Increased values of bacterial abundance were observed throughout summer 2012 and spring–summer 2013 (Fig. 2d), with the 2012 maximum being ~2.5 times lower (up to 13 × 10^5^ cells mL^−1^) than that in 2013 (up to 31.7 × 10^5^ cells mL^−1^).

### Temporal development of gel particles, carbohydrates and amino acids

**Fig. 3.**
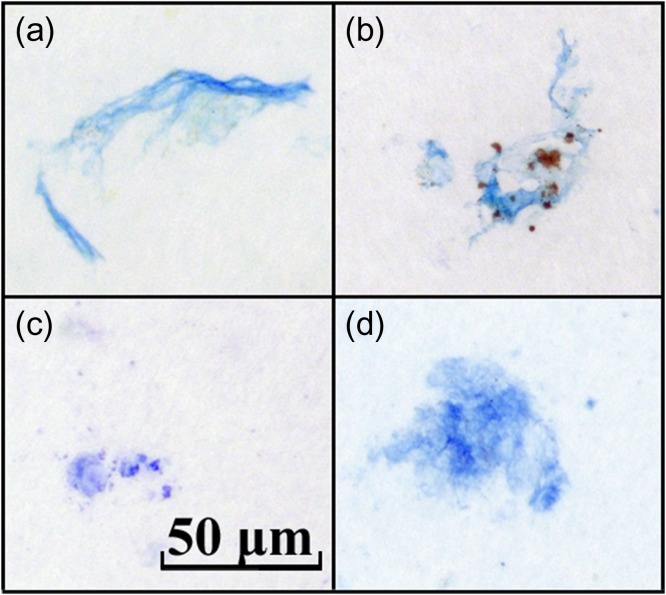
Gel particles visualized by staining: TEP in (**a**) SML; (**b**) SSW, CSP in (**c**) SML; (**d**) SSW.

**Fig. 4.**
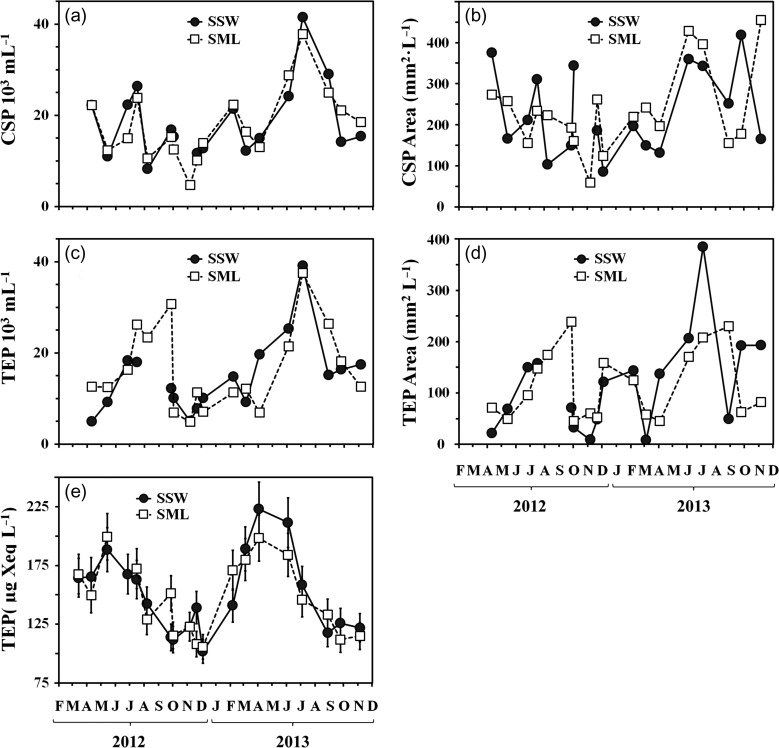
Variations of gel particles in the SSW and SML (**a**) CSP abundance; (**b**) total area of CSP; (**c**) TEP abundance; (**d**) total area of TEP; (**e**) concentrations of TEP measured by the colorimetric method (TEP_col_).

Under the microscope, TEP determined in the SML and in the SSW appeared as small globules, strings or sheets of semitransparent material (Fig. 3a and b). The abundance of TEP (Fig. 4c) ranged from 5 × 10^3^ to 39 × 10^3^ mL^−1^, while their total area (Fig. 4d) ranged from 9 to 388 mm^2^ L^−1^. Both the total area and the abundance of TEP demonstrated elevated values during the summer (May–September) and, to a lesser extent, also during the winter (November–February) periods. The abundance of TEP was significantly correlated with the total area in the SML (*r*^2^ = 0.69, *P* < 0.0001, *df* = 17) and in the SSW (*r*^2^ = 0.86, *P* < 0.0001, *df* = 17). For the SSW, TEP abundance also exhibited a significant correlation with bacterial abundance (*r*^2^ = 0.54, *P* < 0.005, *df* = 17).

Shapes of CSP were similar to those of TEP, however, some CSP clearly appeared as aggregates of smaller particles (Fig. 3c and d). The abundance of CSP (Fig. 4a) ranged from 5 × 10^3^ to 41 × 10^3^ mL^−1^and their total area (Fig. 4b) from 57 to 455 mm^2^ L^−1^, close to the corresponding ranges of TEP. The temporal development of CSP abundance was similar to TEP, with an elevated abundance during the winter and summer periods (the only exception occurred in August 2012). The total area of CSP revealed frequent oscillations, without long periods of higher and lower values. Correlation between the abundance and the total area of CSP was low but still significant (*r*^2^ = 0.35, *P* = 0.01, *df* = 17 in the SML and *r*^2^ = 0.29, *P* = 0.03, *df* = 17 in the SSW). Similar correlations were also found between CSP and bacterial abundances (*r*^2^ = 0.29, *P* = 0.03, *df* = 17 in the SML and *r*^2^ = 0.26, *P* = 0.04, *df* = 17 in the SSW). Furthermore, the abundance of CSP demonstrated a significant temporal coupling to that of TEP, with *r*^2^ = 0.57 (*P* < 0.005, *df* = 16) in the SSW and *r*^2^ = 0.46 (*P* < 0.005, *df* = 16) in the SML.

TEP concentrations (Fig. 4e) determined by the colorimetric method (TEP_col_) ranged from 102 to 223 μg Xeq. L^−1^. Both SML and SSW were enriched in TEP concentration seasonally (March–June), not repeating the winter extreme obtained by microscopy. In the SSW, TEP_col_ were significantly coupled to bacterial abundance throughout the year (*r*^2^ = 0.47, *P* < 0.005, *df* = 17).

**Fig. 5.**
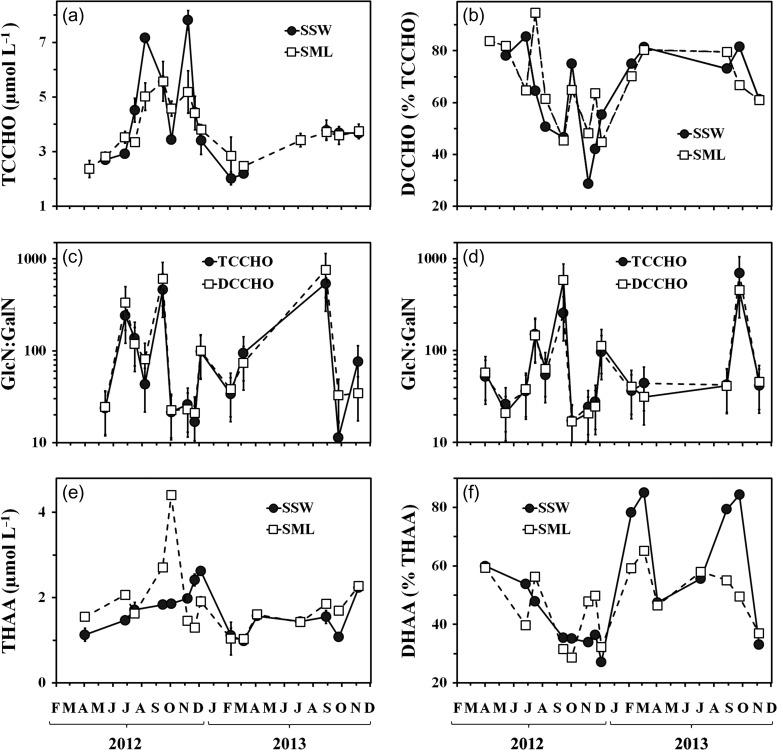
Variations of (**a**) TCCHO concentrations; and (**b**) percentages of dissolved combined carbohydrates (DCCHO) of TCCHO; (**c**) ratio of GlcN to GalN (GlcN:GalN) in the SSW; (**d**) ratio of GlcN to GalN (GlcN:GalN) in the SML; (**e**) THAA concentrations; (**f**) percentages of DHAA of THAA.

TCCHO concentrations (Fig. 5a) varied from 2.0 to 7.8 μmol L^−1^. Dissolved fractions (DCCHO) comprised 29–86% and 45–95% of TCCHO in the SSW and the SML, respectively, with the lower percentages occurring in the course of the elevated carbohydrate abundance from July to December (Fig. 5b). During this period, the DCCHO concentrations in the SSW were directly correlated with Chl *a* (*r*^2^ = 0.77, *P* < 0.01, *df* = 7).

The highest percentage among individual sugars had Glc with up to 81 Mol%, followed by the sum of Man and Xyl with up to 32 Mol%. Fuc, Gal, GlcN and Rha contributed up to 16 Mol% each, while all other sugars were generally less abundant (<4 Mol%). The sugar composition showed large variations among the samples collected at different seasons both in the SSW and in the SML. The most pronounced changes in carbohydrate composition were observed between November 2012 and August 2013, when Mol% of Glc decreased by a factor of 3 and Mol% of deoxysugars (Fuc, Rha), Gal and GlcN increased by a factor of 4 to 7.

Over the study period, the ratio of GlcN to GalN (GlcN:GalN) varied between 11 and 762. On average, GlcN:GalN in TCCHO was slightly higher for the SSW (130 ± 169) than for the SML (110 ± 168) samples. For DCCHO, the difference between SML and SSW was even more pronounced with GlcN:GalN being 163 ± 238 in the SSW and 119 ± 238 in the SML. The most notable differences between SML and SSW were observed in June 2012, March and November 2013 (DCCHO only), when the ratios differed by a factor of 2–9 (Fig. 5c and d).

THAA concentrations ranged from 1.0 to 4.4 μmol L^−1^ (Fig 5e). In general, the contribution of DHAA to THAA was 27–85% in the SSW and 29–65% in the SML (Fig. 5f). The minor percentages of DHAA were determined in autumn (September–December), however, both THAA and DHAA concentrations in the SSW followed the concentrations of Chl *a* over the entire period (*r*^2^ = 0.74 for THAA, and *r*^2^ = 0.39 for DHAA, *P* < 0.05, *df* = 13).

The composition of amino acids was characterized by the dominance of Gly, which contributed up to 35 Mol%. Less abundant were AsX, GlX, Ser and Ala, with the contribution up to 19 Mol% each. Thr comprised up to 11 Mol%, and the other amino acids were more scarce (<8 Mol%). The amino acid composition varied with seasons; however, changes were not as pronounced as those of sugars. Generally, variations in Mol% of the most abundant amino acids, including Thr, varied <2-fold (data not shown).

### Accumulation of organic substances in the SML

**Fig. 6.**
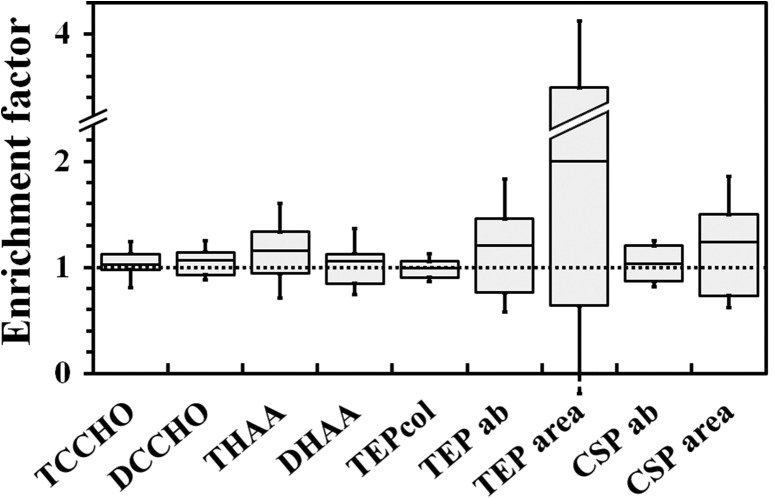
Box and whisker plot of EF calculated for various components of the SML. Each box encloses 50% of the data with the mean value displayed as a line. The extent of each whisker represents the standard deviation of the data.

About 50% of the samples for carbohydrates and DHAA were not enriched significantly in the SML relative to the SSW, having EF between 0.8 and 1.2 (Fig. 6). The other samples showed a higher variability, with maximum EF 1.4 for TCCHO, 1.5 for DCCHO, 2.4 for THAA, 1.9 for DHAA and 1.3 for TEP_col_ concentrations (data not shown). The highest EF was determined for abundances and total areas of gel particles with maximum values of 2.6 and 6.9, respectively, for TEP, and 1.5 and 2.8 for CSP. The results of Mann–Whitney *U*-tests, however, showed that none of the measured parameters was generally enriched in the SML (*P* > 0.2, *df*: between 15 and 18).

No correlation between EF values of all organic components, excluding TCCHO, and wind speed was determined during this study. For TCCHO, EF showed a significant negative linear correlation, albeit with a relatively low coefficient of determination, *r*^2^ = 0.38 (*P* = 0.04, *df* = 11). We also tested for an exponential relationship between the enrichment of TCCHO and the wind speed. The results indicated a slightly better correlation with *r*^2^ = 0.42 (*P* = 0.03, *df* = 11).

The enrichment of each group of compounds, including TCCHO, varied during all three dates of low wind speed detected. Thus, EFs for amino acids and carbohydrates were higher during the calm sampling day in October 2012, but were around unity during the other calm events (March and August 2013). The abundance of TEP conversely showed elevated EF in March and August 2013, whereas it was less than unity in October 2012. Enrichment in CSP was observed only in March 2013.

Table I presents Spearman's rank correlations between organic components in the SML and those in the SSW. Generally, carbohydrates and gel particles showed a significant correlation between both layers, while amino acids did not. Higher correlation coefficients (*r* > 0.70) were determined particularly for TCCHO and TEP_col_ concentrations as well as for the abundances of CSP.

**Table I:** Spearman's rank correlations (*r*) between concentrations of various organic components in the SML and SSW

	*R*	*P*	*N*
TCCHO	0.75	<0.001	14
DCCHO	0.70	<0.01	14
THAA	0.43	0.06	15
DHAA	−0.19	>0.1	15
TEP	0.79	<0.001	18
TEP abundance	0.50	0.02	15
TEP area	0.46	0.03	15
CSP abundance	0.84	<0.001	17
CSP area	0.19	>0.1	17

The pairs of the SML and the SSW data sets compared by Mann–Whitney *U*-tests showed statistically insignificant difference (Mann–Whitney *U*-tests, *P* > 0.5, *df*: between 15 and 18).

**Table II:** The amount of variance (in %) explained by the first principal components (PC1)

	TCCHO	DCCHO	THAA	DHAA
SML	61	41	50	31
SSW	60	36	36	36

### Principal component analysis: trends in sugar and amino acid composition

**Fig. 7.**
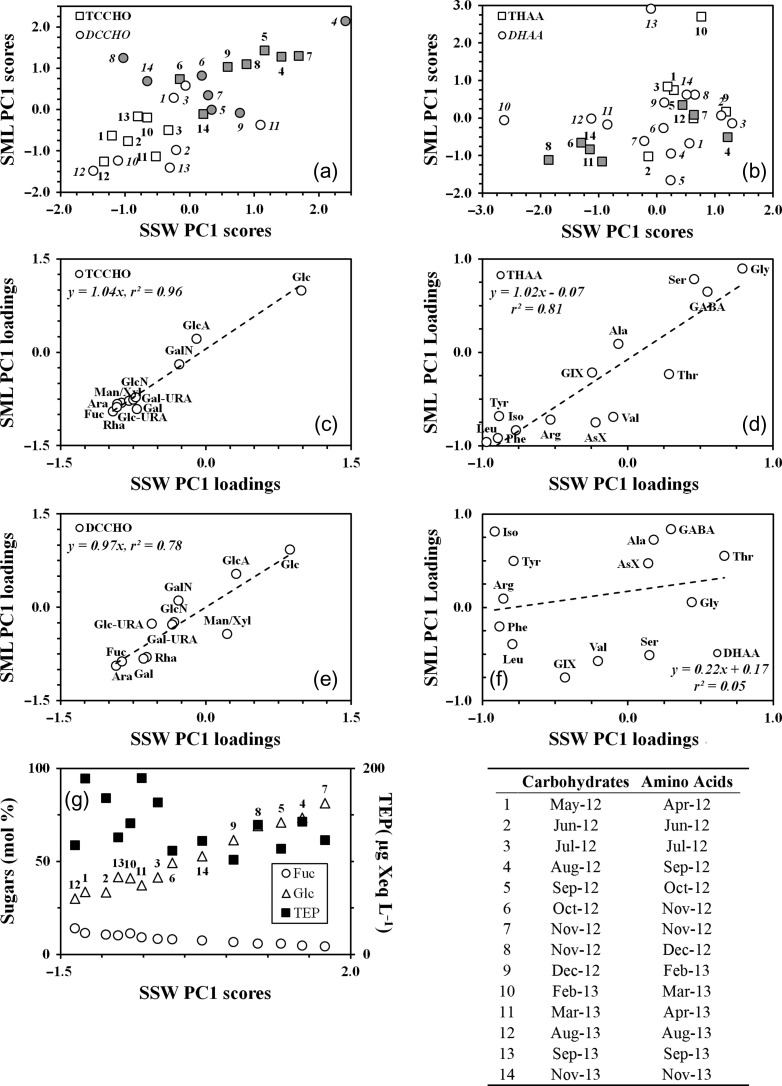
(**a**) The first principal component (PC1) scores for TCCHO and DCCHO in the SML and SSW (4 separate PCA). Shaded data points are regarded to the periods of spring and autumn phytoplankton blooms; (**b**) the first principal component scores for THAA and DHAA in the SML and SSW (four separate PCA). Shaded data points as in Fig. 6a) (**c**) loadings on the PC1s in SML and SSW for TCCHO. Abbreviations: Fuc, fucose; Rha, rhamnose; GalN, galactosamine; Ara, arabinose; GlcN, glucosamine; Gal, galactose; Glc, glucose; Man/Xyl, combined mannose and xylose; GlcA, gluconic acid; GalUA, galacturonic acid and GlcUA, glucuronic acid; (**d**) loadings on the PC1s in SML and SSW for THAA. Abbreviations: Gly, glycine; Ser, serine; Ala, alanine; Tyr, tyrosine; Val, valine; Leu, leucine; Iso, isoleucine; Phe, phenylalanine; AsX, aspartic acid; GlX, glutamic acid; Thr, threonine; Arg, arginine and GABA, γ-aminobutyric acid; (**e**) loadings on the PC1s in SML and SSW for DCCHO. Abbreviations as in Fig. 6c; (**f**) loadings on the PC1s in SML and SSW for DHAA. Abbreviations as in Fig. 6d; (**g**) relative abundances of Fuc and Glc in TCCHO in the SSW and concentrations of TEP_col_ along the PC1; table: Numbers indicate the date of observations for carbohydrates and amino acids.

Figure 7a shows a plot of scores for PC1 for carbohydrates in the SSW and in the SML (Table II). It indicates that the PC1s clearly divided the original data sets in two parts. The first part, represented by positive scores, comprised the data collected over the autumn phytoplankton blooms and the second part, represented by negative scores, typically was formed by the data associated with bacterial bloom in spring–summer periods. In the case of DCCHO, few data points, however, diverged from the division observed. Point 3 (July 2012) in the SML and point 11 (March 2013) in the SSW, for example, associated with the bacterial bloom were located in between the points of the phytoplankton blooms. The possible explanation for such exceptions is that the freshly released DCCHO (i.e. phytoplankton bloom associated) could still quantitatively prevail over that of bacterial bloom, despite low concentrations of Chl *a*.

Loadings of individual sugars of TCCHO on PC1 in SML and SSW are shown in Fig. 7c. Generally, the SML and SSW loadings were similar to each other, with the high positive loading (1.0) provided by Glc and high negative loadings (<−0.7 each) provided by the other sugars, except for GlcA and GalN. The latter showed negligible loadings on PC1.

The PC1s in DCCHO were mainly related to a fewer number of individual sugars, as compared with TCCHO (Fig. 7e). These included Glc, Fuc and Ara, which had absolute loadings higher than 0.9. Gal and Rha were of lower importance, possessing higher loadings in the SML (−0.8 each) than in the SSW (−0.6 each). The other sugars had a medium range of loadings from −0.4 to 0.5.

The overall picture given by PC1 shows the temporal variations in carbohydrate composition, reflecting bloom development. In the course of the phytoplankton blooms, carbohydrates contained higher Mol% of Glc and lower Mol% of Fuc and Ara. In contrast, lower Mol% of Glc and higher Mol% of Fuc and Ara corresponded to the bacterial blooms (Fig. 7g). Each of these periods showed a significant internal variability in carbohydrate composition, with a more or less homogeneous distribution of individual sugar contents.

Although, the PC1 scores for THAA in SML and SSW differed from those in TCCHO, the same division of the data set in two parts remained (Fig. 7b). The first part, formed by a relatively small group of data points having negative scores, was related to phytoplankton blooms. The second part, represented by positive and slightly negative scores, comprised the rest of the data set associated with bacterial blooms. Similar to DCCHO, few data points escaped the division described. Thus, points 4 (September), 5 (October) and 7 (November 2012) of autumn phytoplankton bloom were found in between the points of the elevated bacterial abundances. Again, we explain these exceptions by a quantitative preponderance of amino acids related to bacteria rather than to phytoplankton.

Five individual amino acids Gly, Leu, Iso, Phe and Tyr showed high loadings on the PC1s (Fig. 7d) in THAA. Gly was the only amino acid with a positive loading on the PC1 (>0.8). Leu, Iso, Phe and Tyr were inversely related to Gly, with the loadings lower than −0.7. The samples collected over the phytoplankton blooms, thereby, exhibited different composition from those collected over bacterial blooms, being relatively depleted in Gly and enriched in Leu, Iso, Phe and Tyr.

Contrary to THAA, the PC1 scores for DHAA did not reveal a clear division of the data set based on the association with the microorganism's abundances (Fig. 7f). Furthermore, about two-thirds of the data points composed a relatively compact cluster located near the zero of the PC1 (the plot center). This indicates that the majority of the DHAA samples showed only small differences over the seasonal cycle, representing compositional fluctuations near the average mole percentages of individual amino acids.

## DISCUSSION

### SML enrichment in carbohydrates, amino acids and gel particles

It has previously been shown that different techniques used for SML collection yielded samples that differed in chemical composition and concentration (Estep *et al.*, 1985; Falkowska, 1999; Momzikoff *et al.*, 2004). When such samples are compared with the SSW, an additional uncertainty can arise from differences in SSW sampling (Momzikoff *et al.*, 2004). SSW is often collected at different depths within the first upper meter, potentially displaying large variations in OM concentration when the water column is stratified. EFs are therefore highly sensitive to the sampling procedure, which should be born in mind for quantitative comparisons of different studies.

In our study, sampling was performed with a stainless-steel Garrett-type screen yielding the collection of a relatively thick layer of 200–450 μm (Estep *et al.*, 1985; Henrichs and Williams, 1985; Momzikoff *et al.*, 2004; Obernosterer *et al.*, 2008; Frka *et al.*, 2009). Compared to other sampling techniques that collect thinner layers (<100 μm), screen samples are generally characterized by a higher degree of dilution with underlying water.

In fact, we found that, except for a few samplings, neither carbohydrates nor amino acids were significantly enriched in the SML relative to the SSW. Both groups of compounds generally had the EFs < 1.6, with the sole exception for amino acids which once showed an enrichment of 2.4. In comparison, data obtained by methods sampling thinner SML showed significant enrichment in amino acids (≤7.8, Kuznetsova *et al.* 2004; ≤43.3, Reinthaler *et al.*, 2008; ≤4.6, Engel and Galgani, 2016) and carbohydrates (≤19.4, Gao *et al.*, 2012). This suggests that carbohydrates and amino acids become enriched in a very thin upper part of the SML.

For gel particles, SML enrichment has been frequently shown to exceed that of other compounds, regardless of the method used for sampling (<20, Kuznetsova *et al.*, 2005; Wurl and Holmes, 2008; Wurl *et al.*, 2009; Orellana *et al.*, 2011; Wurl *et al.*, 2011a; Gao *et al.*, 2012; Galgani and Engel, 2013; Engel and Galgani, 2016). This corroborates the idea that the SML is a complex gelatinous layer embedding other organic material (Cunliffe *et al.*, 2013). In the present study, we observed few enrichment “events” in TEP when TEP were quantified with the microscopic technique (EF > 1.8 and >3.3 for the abundance and the total area, respectively); although only a minor enrichment in TEP was detected colorimetrically (EF < 1.3). Such disparity likely originates from the difference between the analytical methods, and may reflect changes in the staining potential of TEP corresponding to varying amounts of acidic and sulfated sugars.

Wind speed is thought to be an important factor controlling the enrichment of particulate material in the SML; it was previously shown that enrichment of both particulate organic carbon and particulate organic nitrogen was negatively correlated with wind speed indicating that increased turbulence can remove particulate material from the SML (Obernosterer *et al.*, 2008). Similar correlations between TEP enrichment and wind speed were reported by Wurl *et al.* (2009; 2011a) and by Engel and Galgani (2016). Our results showe that while the enrichment of total carbohydrates was also inversely related to the wind speed, the enrichment of other microlayer components, including gels, represented a lack of such correlation. Under the conditions of moderate to strong wind speeds, as recorded here (>4.4 m s^−1^, except few calm events), OM seems to be mixed well between the SML and SSW demonstrating no response to a further increase of wind. Liu and Dickhut (1998) revealed that suspended particle concentrations in the SML converged exponentially to those in the SSW with the higher wind. Thereby, they found that the EF of suspended particles was independent of the wind speed higher than 4.0 m s^−1^, similar to our observation.

Without turbulent mixing, the enrichment of SML is generally determined by the surface activity of organic material depending, amongst others, on water pH, temperature, irradiance and bacterial degradation (Kronenberg *et al.*, 2014). The enrichment in surface-active compounds is therefore expected to vary significantly over time. In Boknis Eck, the abundance of surface-active compounds has been previously shown to increase in the uppermost layer of SML (nanolayer) during the summer period (Laß *et al.*, 2013). Although we did not observe any similar trend here, the few periods of low wind speed showed that the EFs of individual components indeed varied over time, indicating that changes in surface-activity occurred within each group of compounds.

The homogenous distribution of organic material at the sea surface obviously leads to a clear statistical link between the SML and SSW, perceived as an apparent coupling of one layer to the other. In fact, significant correlations between the SML and the SSW were calculated here for all groups of compounds except for amino acids. The latter showed a lack of correlation suggesting that the distribution of proteinaceous OM was controlled by factors other than mixing processes. Among the factors that may explain a decoupling of amino acids are heterotrophic consumption by zooplankton (Lee and Cronin, 1984) and by microbial populations (Smith *et al.*, 1992; Kuznetsova and Lee, 2002; Buchan *et al.*, 2014), influencing turn-over rates in the SML and SSW differently (Carlucci *et al.*, 1992), particularly due to a reduced amino acid consumption by bacterioneuston under UV-B (Santos *et al.*, 2012). Similar findings were recently obtained from the highly productive upwelling region off Peru (Engel and Galgani, 2016), where proteinaceous OM also showed weaker correlation between the SML an SSW than other compounds.

Our results indicate a relative homogeneity of the upper water column at the study site since concentrations of organic components were similar between the SML and the SSW. This can partly be explained by SML dilution with underlying water during sampling. However, the prevailing strong winds at our study site could also provide conditions that do not allow the formation of a distinct SML.

### Variations in OM over the annual cycle of pelagic production

**Fig. 8.**
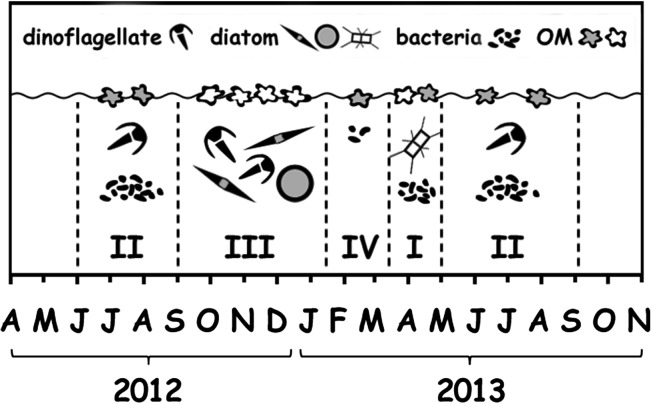
Seasonal pattern of ecosystem development in Boknis Eck. The abundance of phytoplankton, bacteria and concentrations of OM in the SML is shown schematically. The open and filled OM shapes correspond to fresh and degraded material, respectively. For further explanations see the text.

Seasonal fluctuation of biomass and phytoplankton species composition is an annually recurring feature in most temperate coastal systems, including Boknis Eck, where it has been described in detail (Wasmund *et al.*, 2008). According to Wasmund *et al.* (2008), four major stages in the annual cycle of the phytoplankton community can be identified there (Fig. 8). The first stage is associated with the spring bloom dominated by diatom species such as *Chaetoceros*, *Thalassiosira* and *Skeletonema* spp. It is characterized by high biomass build-up subsequently deposited on the sea floor after the bloom termination. The second stage, formed during summer stratification, primarily reflects the growth of flagellates, mainly dinoflagellates such as *Ceratium* spp. and *Dinophysis* spp. Cyanobacteria can also appear although the significance of cyanobacterial micro- compared with picoplankton seems to depend on the inflow from the Central Baltic Sea (Lenz, 1977b; Jochem, 1988). Over this period, the bulk of the production is maintained by remineralization in the euphotic zone caused by zooplankton and heterotrophic bacteria. The latter presumably consist of bacteroidetes and few subgroups of proteobacteria (Stolle *et al.*, 2011). During autumn, phytoplankton blooms associated with a diverse diatom community (*Coscinodiscus* spp., *Pseudo-nitzschia* spp., *Thalassiosira decipiens*, *T. baltica* and others) and dinoflagellates species (mainly *Ceratium* spp.) make up the third stage of plankton community growth. This stage resembles the first one, with similar levels of phytoplankton biomass and rates of sedimentation. Finally, winter dormancy (January–February) with low phytoplankton biomass represents the fourth stage of the cycle.

Based on Chl *a* concentration and bacterial abundance data, we assume that the sequence of bloom stages similar to those described above occurred during the present study (Fig. 8), although with some variation for the first stage. The first stage likely passed over April 2013 characterized by the elevated concentrations of Chl *a*. It seems, however, that phytoplankton biomass was partly recycled responding to an untypical, for this stage increase, bacterial abundance starting in March 2013. The second stages were likely formed over the summer period 2012 and 2013, although the onset of the second stage 2013 cannot be clearly identified due to the early increase of bacterial abundance already mentioned. The third stage seems to have been over September–December 2012, when the elevated concentrations of Chl *a* were recorded. Over February 2013 characterized by minor amount of Chl *a*, the fourth stage likely passed.

Concentrations of carbohydrates and amino acids at the Boknis Eck station varied significantly, but overall agreed well with the range of previously reported data from other coastal locations (Ittekkot *et al.*, 1981; Ittekkot, 1982; Lee and Cronin, 1984; Henrichs and Williams, 1985; Kuznetsova *et al.*, 2004; Görs *et al.*, 2007; Reinthaler *et al.*, 2008; Tsukasaki and Tanoue, 2010; Engel *et al.*, 2012). Generally, both groups of compounds displayed higher concentrations over summer and autumn.

Increased concentrations of carbohydrates and amino acids were reported to be a consistent feature during various natural (Ittekkot *et al.*, 1981; Ittekkot, 1982; Hubberten *et al.*, 1994; Børsheim *et al.*, 1999; Davis and Benner, 2005) and experimental phytoplankton blooms (Biddanda and Benner, 1997; Biersmith and Benner, 1998; Meon and Kirchman, 2001; Børsheim *et al.*, 2005, Engel *et al.*, 2012). Generally, carbohydrate and amino acid concentrations are expected to follow the abundance of phytoplankton as the former account for a large proportion of cellular OM (Hecky *et al.*, 1973; Cowie and Hedges, 1992; Granum *et al.*, 2002). In fact, we found a positive correlation between Chl *a* and THAA concentrations, similar to those reported previously for particulate carbohydrates (Hung *et al.*, 2003), dissolved neutral sugars (Meon and Kirchman, 2001) and dissolved amino acids (Hubberten *et al.*, 1994). At the same time, there was a lack of correlation between Chl *a* and TCCHO concentrations, which could be due to variations in phytoplankton species composition. Different phytoplankton species were shown to exhibit high variability in cellular carbohydrate content (Hecky *et al.*, 1973). Most probably, dinoflagellates and diatom species succeeded each other during the study period, supporting variations in carbohydrate amounts. Furthermore, it was revealed that relative amounts of proteins and carbohydrates in phytoplankton cells showed different seasonal trends, that is, a time shift during the compounds syntheses (Suárez and Marañón, 2003). A similar shift could obviously cause uncoupling of Chl *a* to carbohydrate concentrations.

During this study, dissolved forms generally made up a major proportion of total carbohydrates and amino acids. The exception was found over the third stage, when the proportions of dissolved compounds were lower (<47% for DCCHO, <36% for DHAA). Previous studies showed that the percentages of extracellular release during phytoplankton growth ranged from <14% for diatoms to <43% for dinoflagellates (Lancelot, 1983). Biddanda and Benner (1997) reported similar proportions of dissolved carbohydrates (<35%) during the experimental bloom of the diatom *Skeletonema* sp. Apart from the direct release by phytoplankton, dissolved compounds can also originate from solubilization of particulate OM. The latter is considered to be related to zooplankton grazing, i.e. “sloppy feeding” (Hellebust, 1974; Strom *et al.*, 1997) and bacterial degradation (Buchan *et al.*, 2014). In Boknis Eck, zooplankton are known to be numerous throughout the year, being less abundant during winter (Smetacek *et al.*, 1984). This was indirectly confirmed here by the appreciably high ratio of GlcN to GalN, which indicates the phyto- and zoo-plankton origin of organic material (Benner and Kaiser, 2003).

Both grazing and bacterial degradation can lead to particulate and dissolved OM that is compositionally different from the phytoplankton source. The most common differences were reported to be among the relative abundances of Glc and Gly (Cowie and Hedges, 1996; Müller *et al.*, 1986; Nguyen and Harvey, 1997; Amon *et al.*, 2001; Panagiotopoulos and Sempéré, 2005). In phytoplankton, Gly mostly constitutes the protein complexes of cell walls, whereas Glc is the dominant component of intracellular carbohydrates (Hecky *et al.*, 1973; Cowie and Hedges, 1996). Consumption seems to provide preferential losses of intracellular compared to cell wall material, resulting in OM modification, such as depletion in Glc and enrichment in Gly. Other reported differences include the increase in the relative abundances of deoxysugars (Fuc, Rha), Ara and Gal as well as the amino acids Thr and Ser (Müller *et al.*, 1986; Cowie and Hedges, 1996; Amon *et al.*, 2001; Meon and Kirchman, 2001; Panagiotopoulos and Sempéré, 2007; Davis *et al.*, 2009). However, it is not obvious that these differences are solely attributed to OM modification due to consumption.

Exudates released during phytoplankton blooms can also cause at least transient effects on composition of accumulated OM. It is known that exudates are complex exopolymeric substances (EPS) composed of carbohydrates and proteins, with carbohydrates representing a substantial fraction of the total release (Hoagland *et al.*, 1993; Decho and Herndl, 1995; Myklestad, 1995; Giroldo *et al.*, 2005, Engel *et al.*, 2012; Borchard and Engel, 2015). Several individual sugars, including Fuc, Rha, Ara, Man/Xyl, Gal and Glc are found to compose the bulk of EPS biomass, although their significances seem to vary with the species composition (Table III). Most probably, selected types of EPS containing Ara and deoxysugars participate in the formation of TEP, which were shown to be enriched in Fuc, Rha and to a lesser degree Ara, similar to their EPS precursors (Mopper *et al.*, 1995; Zhou *et al.*, 1998). Thus, one can expect that natural OM mixture would have a relatively high proportions of deoxysugars and Ara when TEP are abundant in the seawater.

**Table III:** Major individual sugars of EPS reported for various phytoplankton species

Species	Rha	Fuc	Man/Xyl	Gal	Glc	Reference
*Chaetoceros spp*	+	+		+		Haug and Myklestad (1976)
*Prasinococcus capsulatus*				+	+	Myklestad (1999)
*Chrysochromulina polylepis*			+	+	+	Myklestad (1999)
*Nitzschia frustulum*	+		+			Allan *et al.* (1972)
*Coscinodiscus nobilis*	+	+	+		+	Percival *et al.* (1980)
*Phaeocystis sp*				+	+	Biersmith and Benner (1998)
*Emiliania huxleyi*		+	+	+	+	Biersmith and Benner (1998)
*Skeletonema costatum*		+	+		+	Biersmith and Benner (1998)
*Synechococcus bacillaris*		+	+	+		Biersmith and Benner (1998)
*Cryptomonas tetrapyrenoidosa*		+	+	+		Giroldo *et al.* (2005)

“+” indicates the individual sugar significance in EPS.

Compositional variations associated with the vernal development of the different bloom stages explained the largest variance in TCCHO, DCCHO and THAA data sets, indicating that both production and consumption processes modify the molecular composition of OM. Examining the first principal component (PC1), we found that such modifications led to compositions included the higher Mol% of Glc and amino acids Leu, Iso, Phe and Tyr on the one side and the higher Mol% of Gly and sugars Fuc, Rha, Ara and Gal on the other side. As the former of these compositions occurred over the first and the third stages, it was primarily characterized by new production. In contrast, the latter of these compositions was generally represented over the second and the fourth stages indicating its association with degradation by consumption. Similar results, derived from OM in sediments (Dauwe and Middelburg, 1998; Dauwe *et al.*, 1999), sinking particles (Sheridan *et al.*, 2002; Goutx *et al.*, 2007) and melted ice floe (Amon *et al.*, 2001), have been interpreted to reflect the extent of material degradation. The cited studies, however, concerned systems where degradation mainly occurred in the absence of autotrophic production. In this study, degraded material was replenished with freshly produced one; therefore, the compositions observed here indicate a mixture of more and less degraded material.

Higher mole percentages of deoxysugars and Ara coincided with increased concentrations of TEP_col_ (Fig. 7g), as well as with higher proportions of TEP_col_ to total carbohydrates (data not shown), suggesting that TEP contributed to material comprised of these sugars. Alternatively, the increase in mole percentages of deoxysugars and Ara could also be caused by the presence of large amounts of degraded material corresponding to a notable bacterial abundance.

Since phytoplankton are suggested to be a primary source of TEP, introducing copious amounts of gelatinous material into the seawater, many previous studies on TEP have focused on periods associated with phytoplankton blooms (Passow, 2002b). The data collected over non-bloom periods are scarce and generally demonstrate a diminished TEP content, particularly related to bacteria and other more specific environmental factors (Passow *et al.*, 2001; Corzo *et al.*, 2005; Radić *et al.*, 2005; Sugimoto *et al.*, 2007; Ortega-Retuerta *et al.*, 2010; Klein *et al.*, 2011). In the present study, the seawater was relatively depleted in gel particles during the autumn phytoplankton blooms, with TEP amounts at the lower end of the range earlier found in surface coastal waters (100–3000 μg Xeq. L^−1^, ≤3.8·10^8^ particles mL^−1^, Passow, 2002b). During the summer periods, we detected increased, but still relatively low amounts of TEP, indicating either low phytoplankton production of TEP, or high loss of TEP due to zooplankton grazing and bacterial degradation. Feeding experiments have shown that the diet of some zooplankton species such as the euphausiid *Euphausia pacifica* and copepod *Calanus pacificus* can partially contain TEP (Passow and Alldredge, 1999; Ling and Alldredge, 2003). The presence of bacteria appears to favor the degradation of TEP and exudates produced by diatom *Skeletonema costatum* (Grossart *et al.*, 2006), *Chaetoceros compressus* (Taylor and Cunliffe, 2017) and the freshwater cryptophyte *Cryptomonas tetrapyrenoidosa* (Giroldo *et al.*, 2005). Alternatively, bacteria release gelatinous material (Grossart, 1999; Stoderegger and Herndl, 1999; Passow, 2002a; Sugimoto *et al.*, 2007; Ortega-Retuerta *et al.*, 2010), and potentially contribute to increased TEP concentrations.

The abundance of CSP was generally similar to that of TEP (Fig. 4a and c), unlike previous findings showing that CSP are more (Long and Azam, 1996; Radić *et al.*, 2006; Engel and Galgani, 2016) or less abundant than TEP (Grossart, 1999; Prieto *et al.*, 2002, Engel *et al.*, 2015). Little is known about the origin and fate of CSP in the ocean (Cisternas-Novoa *et al.*, 2015). In particular, whether CSP are different from TEP or represent the parts of the same particles with different chemical nature is not known so far. Owing to separate staining preceding the measurements, none of the existing evidence on TEP and CSP can be interpreted unambiguously, even though distinct temporal and spatial differences occur for the appearance of each gel. In our study, the abundance of CSP was related to TEP (*r*^2^ = 0.57 in the SML and *r*^2^ = 0.46 in the SSW, *P* < 0.005) suggesting that TEP and CSP dynamics were coupled. A similar finding has previously been reported by Kuznetsova *et al.* (2005), who showed similar total particle volumes for TEP and CSP both in the SML and SSW.

In the present study, both concentration and composition of OM was clearly controlled by the seasonal cycle of pelagic production and consumption. Consequently, the dynamics of OM in the SML reflected seasonal changes in SSW. The varying OM quality may influence the ability to form surface films at the air–sea interface with seasonal change. Future studies are needed to understand if and to what extent OM variations in the SML affect air–sea exchange processes.

## CONCLUSIONS

SML collected at the Boknis Eck Time Series Station in most cases showed slight enrichment in carbohydrates, amino acids and gelatinous material, TEP and CSP. The enrichment of total carbohydrates decreased with the wind speed, supporting the idea that the wind could partially control OM distribution during this study. Higher enrichment of amino acids occasionally coincided with calm wind periods, while some calm periods did not reveal significant enrichment in OM indicating that factors other than wind influenced the SML. Wind induced turbulent mixing, creating conditions for rapid exchange between SML and SSW, is however an important process to balance the loss of OM from the SML. Changes in OM concentration and composition in the SML mainly responded to the annual cycle of plankton production as observed for the water column, originating from both production and consumption processes. Thereby, phytoplankton derived OM was characterized by higher fractions of Glc in the pool of combined carbohydrates and by Leu, Iso, Phe and Tyr in the pool of combined amino acids. More degraded organic material had higher fractions of the amino acid Gly, of the deoxysugars Fuc and Rha, as well as of the neutral sugars Ara and Gal. Apart from degraded material, the higher fractions of deoxysugars and Ara may reflect increased TEP concentrations. CSP occurrence was closely associated with that of TEP, suggesting that temporal and spatial dynamics of both types of gels were coupled at our study site. So far, consequences of compositional change in the SML on air–sea exchange processes, such as gas-exchange, are far from being understood. This study suggests that compositional changes in OM at the air–sea interface are closely related to seasonal variations in the ecology of the underlying water column. Thus, for a better understanding of feed-backs between the ocean and the atmosphere ecological processes and ecosystem changes need to be considered.
